# Real-world analysis of the impact of finerenone on estimated glomerular filtration rate and albuminuria in patients with diabetic kidney disease

**DOI:** 10.1093/ckj/sfaf292

**Published:** 2025-09-17

**Authors:** Kiyomi Ichijo, Ryo Yamaguchi, Hiroyuki Takashima, Hiroki Kobayashi, Takashi Maruyama, Masanori Abe

**Affiliations:** Division of Nephrology, Hypertension and Endocrinology, Department of Medicine, Nihon University School of Medicine, Tokyo, Japan; Division of Nephrology, Hypertension and Endocrinology, Department of Medicine, Nihon University School of Medicine, Tokyo, Japan; Division of Nephrology, Hypertension and Endocrinology, Department of Medicine, Nihon University School of Medicine, Tokyo, Japan; Division of Nephrology, Hypertension and Endocrinology, Department of Medicine, Nihon University School of Medicine, Tokyo, Japan; Division of Nephrology, Hypertension and Endocrinology, Department of Medicine, Nihon University School of Medicine, Tokyo, Japan; Division of Nephrology, Hypertension and Endocrinology, Department of Medicine, Nihon University School of Medicine, Tokyo, Japan

**Keywords:** albuminuria, chronic kidney disease, estimated glomerular filtration rate, finerenone, type 2 diabetes

## Abstract

**Aims:**

Large-scale clinical trials have shown that finerenone reduces the urinary albumin-to-creatinine ratio (UACR) and slows estimated glomerular filtration rate (eGFR) decline, thereby inhibiting a composite cardiovascular and kidney endpoint. However, the efficacy and safety of finerenone in clinical practice remain unknown. This study evaluated eGFR decline and changes in UACR as efficacy endpoints and changes in the serum potassium level as a safety endpoint in patients with diabetic kidney disease (DKD).

**Methods:**

This retrospective observational study was conducted in a real-world clinical setting and included patients with DKD. Eligible patients were those diagnosed with chronic kidney disease stage G1 to G4 who had a UACR of ≥30 mg/gCr while taking a renin–angiotensin system inhibitor and who had initiated finerenone. Endpoints included changes in the eGFR slope, UACR, other urinary biomarkers, laboratory and vital parameters, and adverse events.

**Results:**

The analysis included 120 patients. Finerenone significantly improved the rate of eGFR decline from –4.99 (–5.75, –4.23) to –0.59 (–1.24, 0.07) mL/min/1.73 m^2^/year (*P* < .0001). UACR also decreased significantly after finerenone treatment from 908 to 487 mg/gCr (*P* < .0001). Finerenone improved the eGFR slope across all baseline eGFR and albuminuria categories. The rate of eGFR decline improved regardless of whether sodium–glucose cotransporter 2 inhibitor therapy was used concomitantly. Symptomatic hypotension, acute kidney injury and hyperkalemia leading to drug discontinuation were uncommon.

**Conclusions:**

This real-world analysis suggests that finerenone may improve the eGFR slope in patients with DKD without causing significant hyperkalemia, regardless of baseline eGFR and albuminuria values.

## GRAPHICAL ABSTRACT

**GRAPHICAL ABSTRACT:**
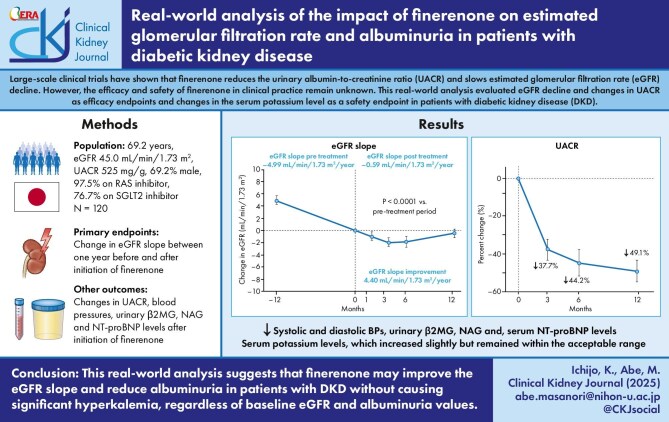

KEY LEARNING POINTS **What was known:**

- Finerenone selectively inhibits mineralocorticoid receptor overactivation, while maintaining the serum potassium level within acceptable limits.
- Large-scale clinical trials have shown that finerenone reduces the urinary albumin-to-creatinine ratio and slows estimated glomerular filtration rate (eGFR) decline.
- In the FIDELIO-DKD trial, finerenone reduced the risk of the primary renal composite endpoint by 18% compared with placebo.

**This study adds:**

- Finerenone improved the eGFR slope across all baseline eGFR and albuminuria categories.
- Finerenone improved the rate of GFR decline regardless of whether it was administered concomitantly with an sodium–glucose cotransporter 2 inhibitor.
- Finerenone reduced urinary β_2_-microglobulin levels as well as the N-terminal pro-brain natriuretic peptide levels in patients with diabetic kidney disease (DKD).

**Potential impact:**

- This real-world analysis provides evidence that finerenone may improve the eGFR slope in DKD without causing significant hyperkalemia, regardless of baseline eGFR and albuminuria values.
- Finerenone may have a protective effect on not only the kidneys but also the heart in patients with DKD.
- Finerenone is a useful treatment option for patients with DKD in real-world settings.

## INTRODUCTION

Until 2019, renin–angiotensin–aldosterone system blockade with angiotensin-converting enzyme (ACE) inhibitors or angiotensin receptor blockers (ARBs) was the only treatment available for diabetic kidney disease (DKD) [1]. However, the CREDENCE (Canagliflozin and Renal Events in Diabetes with Established Nephropathy Clinical Evaluation) trial demonstrated that sodium–glucose cotransporter 2 (SGLT2) inhibitors are effective in slowing the progression of DKD [2]. This finding has led to significant advances in the management of chronic kidney disease (CKD) in patients with type 2 diabetes. A number of clinical trials have established the cardiovascular and renal benefits of SGLT2 inhibitors and their role in the management of patients who have CKD and albuminuria [3, 4].

However, in Japan, many patients develop renal impairment as a result of DKD and require kidney replacement therapy for end-stage kidney disease (ESKD) [5, 6]. Despite the availability of these treatments, patients with DKD still have a significant residual risk of cardiovascular and renal events [7]. Residual albuminuria after treatment with an SGLT2 inhibitor is an independent risk factor for these events [8, 9].

Recent clinical trials have shown that finerenone, a nonsteroidal mineralocorticoid receptor antagonist (MRA), slows CKD progression in patients with type 2 diabetes when combined with renin–angiotensin system (RAS) inhibitor therapy [10, 11]. Finerenone selectively inhibits mineralocorticoid receptor (MR) overactivation, which promotes inflammation and fibrosis (the major causes of progression of CKD), while maintaining the serum potassium level within acceptable limits, which is a known concern with steroidal MRAs [12]. Although use of MRAs for DKD has been recommended, the long-term efficacy and safety of finerenone has not been confirmed in Japanese patients with DKD. Therefore, in this study, we investigated changes in estimated glomerular filtration rate (eGFR), the urinary albumin-to-creatinine ratio (UACR) and the serum potassium level over 1 year before and after initiation of finerenone in patients with DKD.

## MATERIALS AND METHODS

### Study design and participants

The retrospective observational study was conducted in a real-world clinical setting involving patients with DKD. Patients with DKD who were started on finerenone 10 mg or 20 mg at Nihon University Itabashi Hospital between April 2023 and April 2024 were identified. The following inclusion criteria were applied: age 18 years or older; diagnosis of type 2 diabetes and CKD; started on finerenone between July 2022 and July 2023; a UACR of ≥30 mg/gCr; an eGFR of ≥25 mL/min/1.73 m^2^; availability of eGFR and UACR data for ≥12 months before starting on finerenone; and a follow-up period of ≥12 months after initiating finerenone. Patients could still be included if they had been taking a maximum tolerated dose of a RAS inhibitor for ≥6 months. Patients who had previously undergone dialysis or kidney transplantation were excluded.

The study was approved by the Ethics Committee of Nihon University Itabashi Hospital (approval number: RK-250408-03) and registered in the University Hospital Medical Information Network Clinical Trial Registry (UMIN000058718). Patient privacy was ensured by anonymizing the data in compliance with the Declaration of Helsinki.

### Dose titration of finerenone

Finerenone was initiated and titrated primarily according to the protocol used in the FIDELIO-DKD (Finerenone in Reducing Kidney Failure and Disease Progression in Diabetic Kidney Disease) trial [10]. The starting dose was based on the eGFR and serum potassium concentration at baseline. Patients with an eGFR of <60 mL/min/1.73 m^2^ were started on 10 mg once daily and those with an eGFR of ≥60 mL/min/1.73 m^2^ were started on 20 mg once daily. At initiation of finerenone, all patients had serum potassium levels below 4.8 mmol/L. Dose titration and adjustment was performed after 1 month according to protocol. If the serum potassium level was ≤4.8 mmol/L, the dose was maintained at 20 mg once daily. For patients on 10 mg once daily, the dose was increased to 20 mg once daily if eGFR had not decreased by ≥30% from the previous measurement. The current dose of finerenone was maintained if serum potassium was 4.9–5.5 mmol/L, discontinued if it was >5.5 mmol/L and resumed at 10 mg once daily if it decreased to ≤5.0 mmol/L.

### Data collection

Serum creatinine (sCr) and potassium levels were measured at baseline and at Months 1, 3, 6 and 12 after starting on finerenone. Hemoglobin, serum urea nitrogen, total protein, albumin, sodium, uric acid, total cholesterol, high-density lipoprotein cholesterol, low-density lipoprotein cholesterol and triglyceride levels were measured by routine clinical chemistry procedures using commercially available assay kits. N-terminal pro-brain natriuretic peptide (NT-proBNP) levels were measured by the electrochemiluminescence immunoassay method. eGFR was calculated according to the following formula for Japanese patients [13]: eGFR (mL/min/1.73 m^2^) = 194 × sCr^−1.094^ × age^−0.287^ (× 0.739 for women).

Urinary albumin excretion was assessed by measuring the UACR. Urinary albumin levels were measured by the immunoturbidimetric assay. Urinary β_2_-microglobulin (β_2_MG) and N-acetyl-β-D-glucosaminidase (NAG) concentrations were measured by latex agglutination and colorimetry, respectively, in the same urine sample.

Use of concomitant medications, such as antihypertensive agents, antidiabetic drugs, statins and uric acid–lowering drugs, was also recorded. Longitudinal eGFR and UACR data were obtained from 12 months before to 12 months after the start of finerenone treatment.

### Outcome measure

The primary outcome was the change in slope of the eGFR before and after treatment. Secondary outcomes included changes in UACR, systolic blood pressure, diastolic blood pressure, urinary NAG and β2MG after treatment. The primary safety outcome measure was the change in serum potassium level, which was measured using an ion-selective electrode method. Subgroup analyses were performed to determine whether the effects of finerenone on the slope of the eGFR differ depending on whether SGLT2 inhibitor and glucagon-like peptide-1 receptor agonist therapy were used concomitantly.

All variables were assessed at baseline and at the end of the study. Efficacy variables were analyzed in all subjects. Subjects could be withdrawn in the event of allergy or intolerance to the drug, if either the serum transaminase or creatine kinase concentration increased to more than 2× the upper limit of normal, or following an event that, in the investigator’s opinion, might have posed a risk to the patient or confounded the results of the study. At each visit, subjects were questioned about compliance with diet and medications, any concomitant medications and adverse events (AEs). Safety assessments were performed throughout the study. AEs were graded as mild, moderate, or severe. Serious AEs were defined as medical events that resulted in death, hospitalization, or significant disability or incapacity.

### Statistical analysis

Data are expressed as the mean ± standard deviation, mean [95% confidence interval (CI)], or median [interquartile range (IQR)] as appropriate. Continuous variables were compared between groups using the Student’s *t*-test or the Mann–Whitney U test, and categorical variables were compared using the χ^2^ test or Fisher’s exact test. Subgroup analyses were performed according to eGFR and albuminuria categories at baseline. Changes in UACR, serum potassium concentration, systolic and diastolic blood pressures, urinary β2MG, NAG and NT-proBNP levels were performed as supplementary analyses to assess the potential impact of finerenone. Changes in UACR were compared according to whether patients received a final dose of finerenone of 10 mg/day or 20 mg/day. Missing values were replaced by multiple imputation methods. All statistical analyses were performed using JMP^Ⓡ^ software version 14 (SAS Institute Inc., Cary, NC, USA). A *P*-value of <.05 was considered statistically significant.

## RESULTS

### Baseline characteristics

Data for the 120 patients who met the inclusion criteria were analyzed. Their baseline characteristics are listed in Table 1. The mean age was 69.2 ± 12.4 years, and 69.2% were male. The median eGFR was 45.0 mL/min/1.73 m^2^ (IQR 31.7, 57.8), and the most common CKD stage was G3b (29.2%), followed by G3a (27.5%), G1 + 2 (22.5%) and G4 (20.8%). The median UACR was 525 mg/g (145, 1090), with 36.7% and 63.3% categorized as A2 and A3, respectively. RAS inhibitors were taken by 97.5% of the patients and SGLT2 inhibitors by 76.7%. The initial daily dose of finerenone was 20 mg in 22.5% of the patients and 10 mg in 77.5%, with a mean daily dose of 12.3 ± 4.2 mg. The final mean daily dose was 19.0 ± 3.0 mg, with 90.0% of the patients receiving 20 mg daily.

**Table 1:** Baseline characteristics of the study participants.

Variable	Total population (*n* = 120)
Age, years	69.2 ± 12.4
Male sex, *n* (%)	83 (69.2)
Body mass index, kg/m^2^	25.4 ± 5.3
History of CVD, *n* (%)	26 (21.7)
Ischemic heart disease	12 (10.0)
Stroke	6 (5.0)
Heart failure	10 (8.3)
PAD	2 (1.7)
Systolic BP, mmHg	133 ± 11
Diastolic BP, mmHg	78 ± 12
Heart rate, bpm	80 ± 16
HbA_1c_, %	6.3 ± 0.6
Total cholesterol, mg/dL	192 ± 33
HDL-C, mg/dL	49 (42, 63)
LDL-C, mg/dL	104 ± 21
Triglycerides, mg/dL	149 (111, 211)
Serum potassium, mmol/L	4.2 ± 0.3
eGFR, mL/min/1.73 m^2^	45.0 (31.7, 57.8)
eGFR category, *n* (%)	
G1 + G2, ≥60	27 (22.5)
G3a, 45 to <60	33 (27.5)
G3b, 30 to <45	35 (29.2)
G4, <30	25 (20.8)
UACR, mg/gCr	525 (145, 1090)
A2, 30 to <300	44 (36.7)
A3, ≥300	76 (63.3)
Medications, *n* (%)	
RAS inhibitors	117 (97.5)
ARBs	113 (94.2)
ACE inhibitors	4 (3.3)
None	3 (2.5)
Calcium channel blockers	82 (68.3)
Diuretics	15 (12.5)
β-blockers	35 (29.2)
SGLT2 inhibitors	92 (76.7)
DPP-4 inhibitors	62 (51.7)
GLP-1 receptor agonists	10 (8.3)
Metformin	32 (26.7)
Insulin	10 (8.3)
Statins	64 (53.3)
Fibrates	18 (15.0)
Antihyperuricemic agents	58 (48.3)
ESAs or HIF-PH inhibitors	6 (5.0)

Data are presented as mean ± standard deviation, median (interquartile range) or *n* (%).

ACE, angiotensin-converting enzyme; ARB, angiotensin receptor blocker; BP, blood pressure; CVD, cardiovascular disease; DPP-4, dipeptidyl peptidase-4; ESAs, erythropoiesis-stimulating agents; GLP-1, glucagon-like peptide-1; HbA_1c_, glycated hemoglobin; HDL-C, high-density lipoprotein cholesterol; HIF-PH, hypoxia-inducible factor prolyl-hydroxylase; LDL-C, low-density lipoprotein cholesterol; PAD, peripheral artery disease.

### Changes in eGFR before and after finerenone initiation

**Figure 1:**
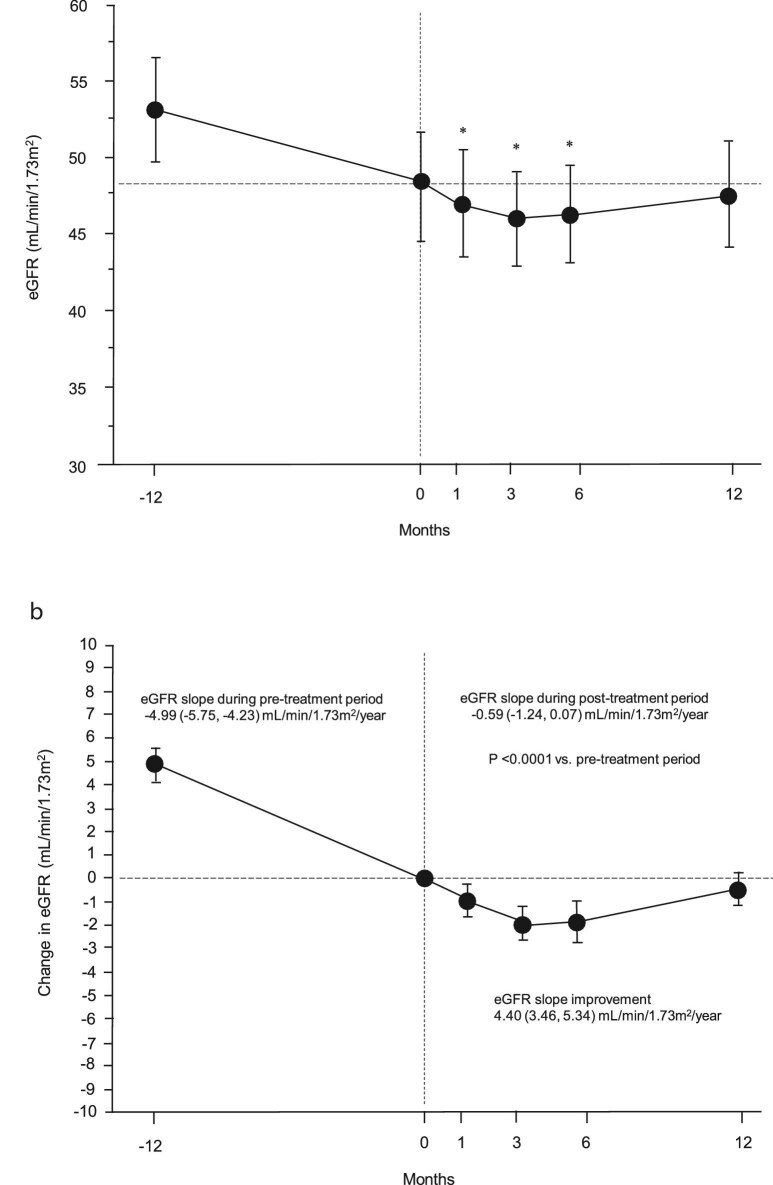
Changes in eGFR during the study period. (**a**) Longitudinal change in mean eGFR before and after finerenone initiation. ^*^P < .0001 vs baseline. (**b**) Longitudinal change in eGFR before and after finerenone initiation, with the mean eGFR at Month 0 centered to 0. The eGFR slope improved from –4.99 (95% CI –5.75, –4.23) mL/min/1.73 m^2^/year during the pre-treatment period to –0.59 (–1.24, 0.07) mL/min/1.73 m^2^/year after finerenone initiation. The improvement in eGFR slope represented 4.40 (3.46, 5.34) mL/min/1.73 m^2^/year.

The changes in eGFR before and after finerenone initiation are shown in Fig. 1a. The mean eGFR (95% CI) 1 year prior to initiating finerenone was 53.1 (49.4, 56.7) mL/min/1.73 m^2^, but had decreased to 48.1 (44.5, 51.6) mL/min/1.73 m^2^ at the time of finerenone initiation (*P* < .0001). At 1 month after finerenone initiation, the mean eGFR decreased significantly compared with baseline to 47.0 (43.6, 50.5) mL/min/1.73 m^2^ and declined further to 45.9 (42.6, 49.3) mL/min/1.73 m^2^ at 3 months (both *P* < .0001). However, after 12 months of finerenone treatment, the mean eGFR had recovered to 47.5 (44.0, 50.9) mL/min/1.73 m^2^ and was not significantly different from the baseline value. The changes in mean eGFR before and after finerenone initiation are shown in Fig. 1b, with the baseline eGFR centered at 0. The decline in the eGFR slope over the 12 months before starting on finerenone was –4.99 (–5.75, –4.23) mL/min/1.73 m^2^/year, which improved to –0.59 (–1.24, 0.07) mL/min/1.73 m^2^/year after 12 months, representing a difference of 4.40 (3.46, 5.34) mL/min/1.73 m^2^/year (*P* < .0001).

### Changes in UACR before and after finerenone initiation

**Figure 2:**
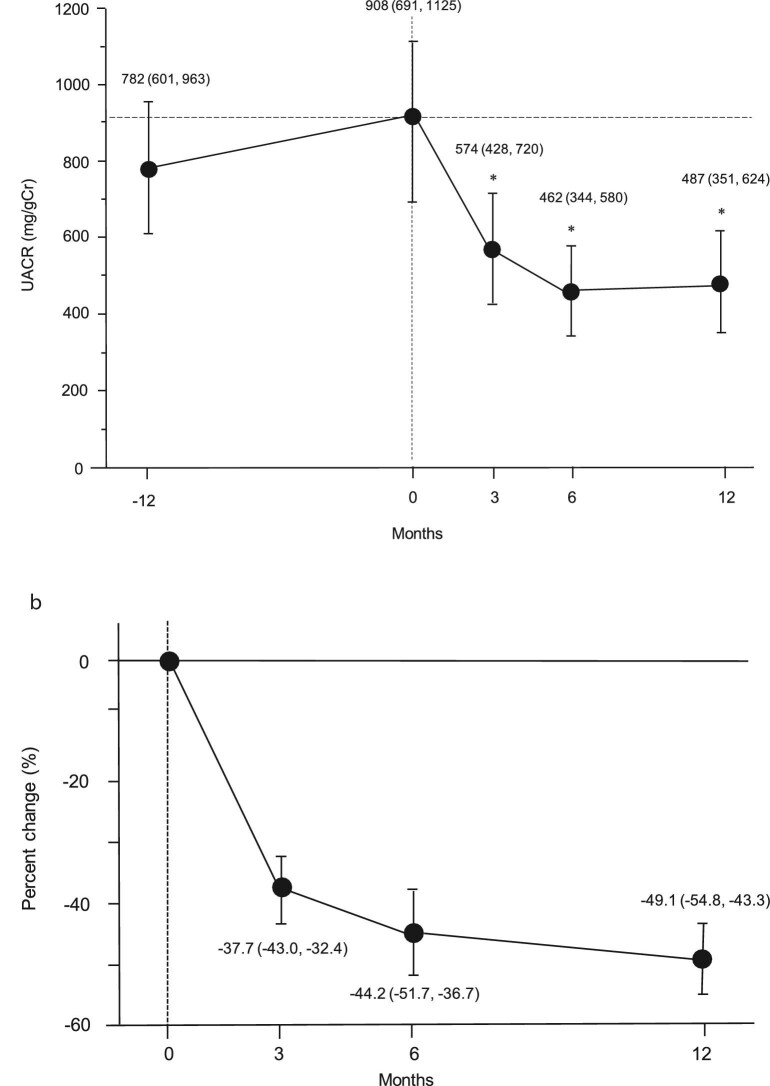
Changes in UACR during the study period. (**a**) Longitudinal changes in UACR before and after finerenone initiation. ^*^P < .0001 vs baseline. (**b**) Percent changes in UACR from baseline. Data are expressed as the mean (95% CI).

The changes in UACR are shown in Fig. 2a. The mean UACR (95% CI) 1 year before finerenone initiation was 782 (601, 963) mg/gCr. However, it had increased significantly to 908 (691, 1125) mg/gCr at the time of finerenone initiation (*P* < .0001). After 3 months of treatment, there was a significant decrease in the mean UACR to 574 (428, 720) mg/gCr compared with baseline (*P* < .0001). Finerenone gradually reduced the UACR over time, with the most significant reduction observed after 6 months. After 12 months, there was a mean % reduction of –49.1% (–54.8%, –43.3%) from baseline (Fig. 2b).

### Subgroup analysis by baseline eGFR and albuminuria category and final daily dosage

**Figure 3:**
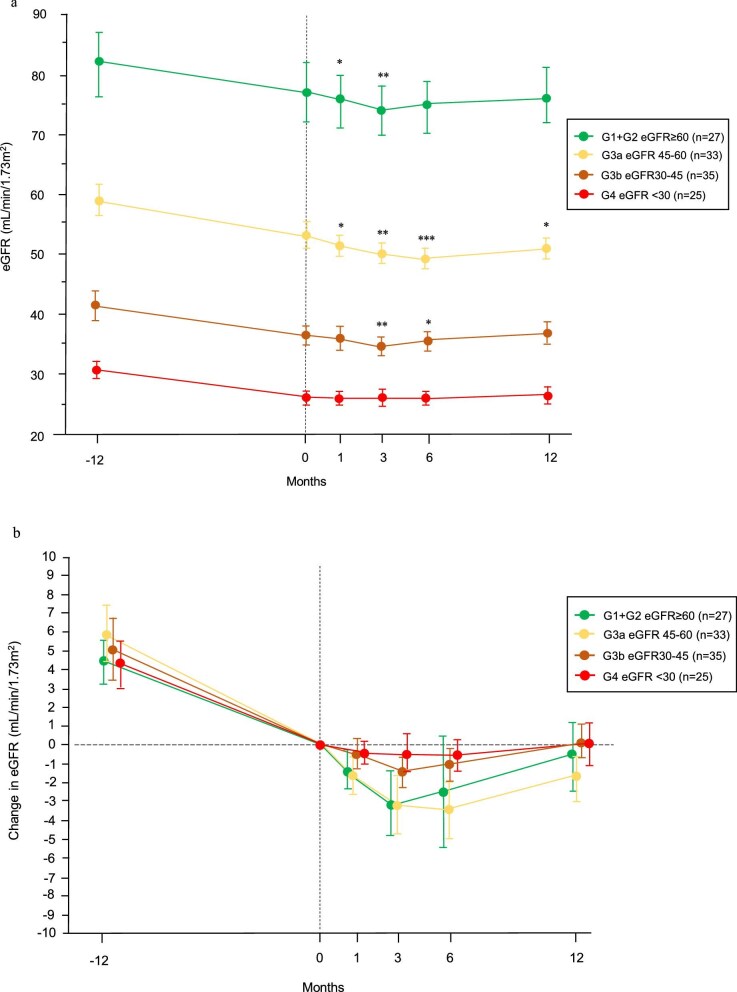
Changes in eGFR during the study period according to baseline eGFR category. (**a**) Longitudinal changes in eGFR before and after finerenone initiation. Data are expressed as the mean (95% CI). ^*^P < .01, ^**^P < .001, ^***^P < .0001 vs. baseline. (**b**) Longitudinal changes in eGFR before and after finerenone initiation, with the mean eGFR at Month 0 centered to 0. In the G1 + G2 group, the eGFR slope improved from –4.42 (–5.64, –3.21) mL/min/1.73 m^2^/year during the pre-treatment period to –0.71 (–2.59, 1.17) mL/min/1.73 m^2^/year after finerenone initiation. The improvement in eGFR slope was 3.71 (1.72, 5.71) mL/min/1.73 m^2^/year (*P* = .007). In the G3a group, the eGFR slope improved from –5.99 (–7.63, –4.34) mL/min/1.73 m^2^/year during the pre-treatment period to –1.80 (–3.02, –0.57) mL/min/1.73 m^2^/year after finerenone initiation. The improvement in eGFR slope was 4.19 (2.38, 5.99) mL/min/1.73 m^2^/year (*P* = .0002). In the G3b group, the eGFR slope improved from –4.99 (–6.70, –3.28) mL/min/1.73 m^2^/year during the pre-treatment period to 0.15 (–0.84, 1.14) mL/min/1.73 m^2^/year after finerenone initiation. The improvement in eGFR slope was 5.14 (3.38, 6.89) mL/min/1.73 m^2^/year (*P* < .0001). In the G4 group, the eGFR slope improved from –4.92 (–5.54, –3.03) mL/min/1.73 m^2^/year during the pre-treatment period to –0.11 (–1.22, 1.34) mL/min/1.73 m^2^/year after finerenone initiation. The improvement in eGFR slope was 4.40 (2.32, 6.48) mL/min/1.73 m^2^/year (*P* < .0001).

Figure 3a shows the decline in the eGFR slope over time according to the eGFR category at baseline. The changes in eGFR before and after finerenone initiation are shown in Fig. 3b, with baseline eGFR centered at 0. In the G1 + G2 group, eGFR decreased significantly at 1 and 3 months compared with baseline, but no significant differences were observed from 6 months onwards. In the G3a group, the decline in eGFR continued until 6 months, but improvement was observed by 12 months. In the G3b group, the greatest decline in eGFR occurred at 3 months, and baseline values were recovered after 12 months. In the G4 group, the degree of acute drop in eGFR was gradual, and the slope of eGFR was flat throughout the study period. In all eGFR categories, the rate of decline in eGFR after 12 months was significantly lower than the rate during the 12 months before finerenone treatment (Table 2).

**Table 2:** Effect of finerenone on changes in the slope of the eGFR stratified by category of eGFR at baseline.

eGFR category (mL/min/1.73 m^2^)	*N*	Pre-slope	Post-slope	Difference (95% CI)	*P*-value
G1 + G2 (≥60)	27	–4.42 (–5.64, –3.21)	–0.71 (–2.59, 1.17)	3.71 (1.72, 5.71)	.0007
G3a (45 to <60)	33	–5.99 (–7.63, –4.34)	–1.80 (–3.02, –0.57)	4.19 (2.38, 5.99)	.0002
G3b (30 to 45)	35	–4.99 (–6.70, –3.28)	0.15 (–0.84, 1.14)	5.14 (3.38, 6.89)	<.0001
G4 (<30)	25	–4.29 (–5.54, –3.03)	0.11 (–1.22, 1.34)	4.40 (2.32, 6.48)	<.0001

Data are presented as mean (95% confidence interval).

**Figure 4:**
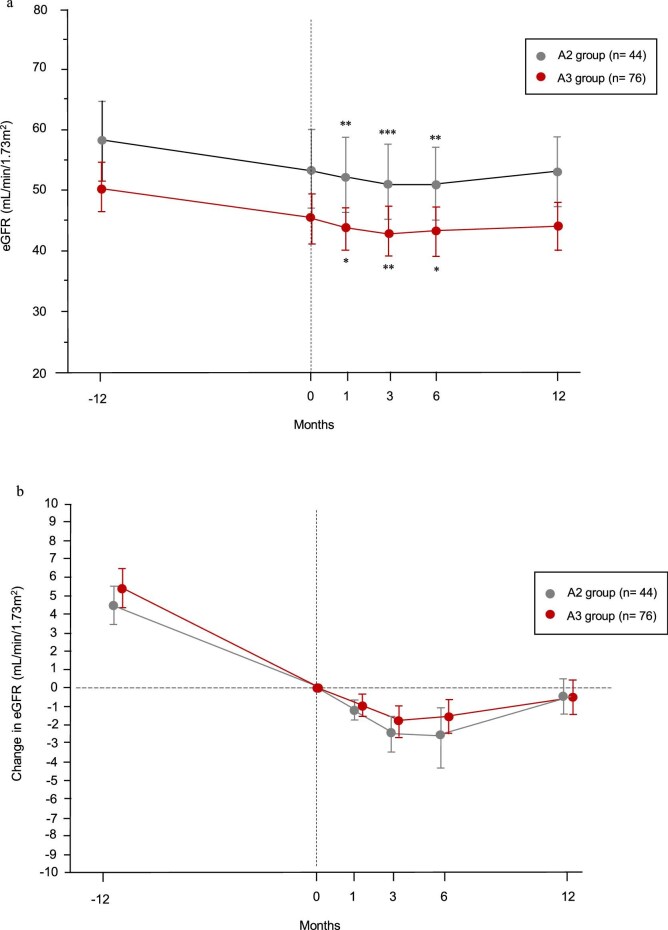
Changes in eGFR during the study period according to baseline albuminuria category. (**a**) Longitudinal changes in eGFR before and after finerenone initiation. Data are expressed as the mean eGFR (95% CI). ^*^P < .01, ^**^P < .001, ^***^P < .0001 vs baseline. (**b**) Longitudinal changes in eGFR before and after finerenone initiation, with the mean eGFR at Month 0 centered to 0. In the A2 group, the eGFR slope improved from –4.39 (–5.45, –3.32) mL/min/1.73 m^2^/year during pre-treatment to –0.47 (–1.52, 0.57) mL/min/1.73 m^2^/year after finerenone initiation. The improvement in eGFR slope was 3.91 (2.58, 5.22) mL/min/1.73 m^2^/year (*P* < .0001). In the A3 group, the eGFR slope improved from –5.34 (–6.36, –4.32) mL/min/1.73 m^2^/year during pre-treatment to –0.65 (–1.50, 0.20) mL/min/1.73 m^2^/year after finerenone initiation. The improvement in eGFR slope was 4.49 (3.40, 5.98) mL/min/1.73 m^2^/year (*P* < .0001).

Changes in eGFR by baseline albuminuria category are shown in Fig. 4a. The changes in eGFR before and after finerenone initiation in groups A2 and A3 are shown in Fig. 4b, with baseline eGFR centered at 0. In group A2, eGFR declined the most at 6 months, but returned to baseline values after 12 months. In group A3, eGFR declined the most at 3 months, but returned to baseline values after 12 months. In both the A2 and A3 groups, the rate of decline in eGFR after 12 months was significantly slower than the rate during the 12 months before finerenone treatment (Table 3).

**Table 3:** Effect of finerenone on changes in the eGFR slope when stratified by baseline albuminuria category.

UACR category (mg/gCr)	*n*	Pre-slope	Post-slope	Difference (95% CI)	*P*-value
A2 (30 to 299)	44	–4.39 (–5.45, –3.32)	–0.47 (–1.52, 0.57)	3.91 (2.35, 5.46)	<.0001
A3 (≥300)	76	–5.34 (–6.36, –4.32)	–0.65 (–1.50, 0.20)	4.69 (3.51, 5.87)	<.0001

Data are presented as mean (95% confidence interval).

When comparing the 10 mg group and the 20 mg group based on final dose of finerenone, no significant difference was observed in the decline in eGFR slope between the two groups. However, the mean % reduction rate in UACR after 12 months was significantly lower in the 10 mg group than in the 20 mg group [20.1% (95% CI –51.8%, 9.81%) vs –52.2% (–57.5%, –46.8%); *P* = .001].

### Subgroup analysis according to concomitant use of SGLT2 inhibitor therapy

**Figure 5:**
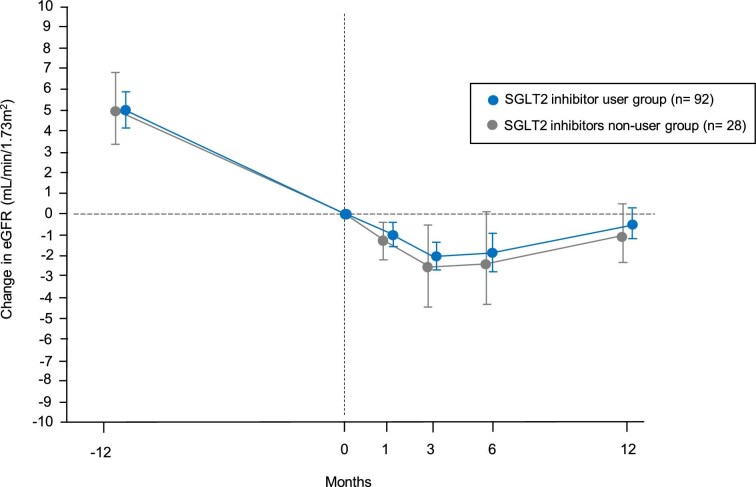
Longitudinal changes in eGFR before and after finerenone initiation according to whether an SGLT2 inhibitor was used concomitantly. This figure shows the mean longitudinal changes in eGFR (95% CI) before and after finerenone initiation, with the mean eGFR at Month 0 centered at 0. In the group receiving concomitant SGLT2 inhibitor therapy, the eGFR slope improved from –5.00 (–5.86, –4.13) mL/min/1.73 m^2^/year during pre-treatment to –0.43 (–1.16, 0.30) mL/min/1.73 m^2^/year after finerenone initiation. The improvement in eGFR slope was 4.57 (3.49, 5.64) mL/min/1.73 m^2^/year (*P* < .0001). In the group that did not receive concomitant SGLT2 inhibitor therapy, the eGFR slope improved from –4.96 (–6.95, –3.32) mL/min/1.73 m^2^/year during pre-treatment to –1.09 (–2.57, 0.38) mL/min/1.73 m^2^/year after finerenone initiation. The improvement in eGFR slope was 3.86 (1.80, 5.92) mL/min/1.73 m^2^/year (*P* = .0006).

Figure 5 shows the changes in eGFR before and after finerenone treatment according to whether an SGLT2 inhibitor was used concomitantly. In both groups, eGFR declined most at Month 3. In the group that used an SGLT2 inhibitor, the mean decline in eGFR slope over the 12 months before finerenone treatment was –5.00 (95% CI –5.86, –4.13) mL/min/1.73 m^2^/year, which improved to –0.43 (–1.16, 0.30) mL/min/1.73 m^2^/year after 12 months, representing a difference of 4.57 (3.49, 5.64) mL/min/1.73 m^2^/year (*P* < .0001). In the group that did not use an SGLT2 inhibitor, the slope of decline in eGFR over the 12 months before finerenone treatment was –4.96 (–6.95, –3.32) mL/min/1.73 m^2^/year, which improved to –1.09 (–2.57, 0.38) mL/min/1.73 m^2^/year after 12 months, representing a difference of 3.86 (1.80, 5.92) mL/min/1.73 m^2^/year (*P* = .0006). However, there was no statistically significant difference in the eGFR slope between the two groups (*P* = .811).

### Changes in kidney-related parameters and vital signs

Changes in kidney-related parameters and vital signs are listed in Table 4. Systolic and diastolic blood pressures decreased significantly between baseline and 12 months after finerenone treatment. There was no significant change in the heart rate. Urinary β2MG and NAG were significantly decreased after finerenone treatment. NT-proBNP levels decreased significantly between baseline and the end of the study.

**Table 4:** Changes in parameters and vital signs at baseline and at the study end.

Variable	Baseline	12 months	*P*-value
Systolic BP, mmHg	133.1 ± 11.1	130.4 ± 12.0	.008
Diastolic BP, mmHg	78.4 ± 11.9	77.0 ± 11.9	.002
Heart rate, bpm	79.6 ± 15.3	78.7 ± 14.9	.086
Hemoglobin, g/dL	13.9 ± 1.5	13.9 ± 1.9	.396
HbA_1c_, %	6.3 ± 0.6	6.3 ± 0.6	.571
Serum creatinine, mg/dL	1.26 ± 0.48	1.27 ± 0.50	.473
eGFR, mL/min/1.73 m^2^	48.1 ± 19.5	47.5 ± 19.3	.704
Serum urea nitrogen, mg/dL	22.7 ± 8.7	22.5 ± 9.5	.605
Uric acid, mg/dL	5.5 ± 1.2	5.5 ± 1.1	.705
Sodium, mmol/L	142 ± 2	141 ± 2	.269
Potassium, mmol/L	4.23 ± 0.34	4.34 ± 0.38	.0004
Urinary β2MG, median, μg/L	685 (330, 2014)	399 (205, 1044)	.007
Urinary NAG, IU/L	9.8 ± 7.4	7.3 ± 5.1	<.0001
NT-proBNP, pg/mL	140 (65, 328)	114 (51, 235)	.0008

Data are presented as mean ± standard deviation or median (interquartile range).

BP, blood pressure; HbA_1c_, glycated hemoglobin.

### Changes in serum potassium levels and safety

**Figure 6:**
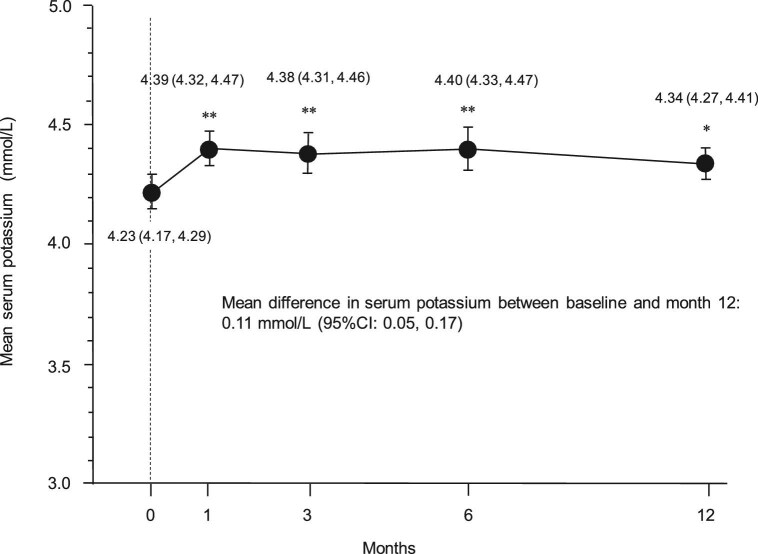
Longitudinal changes in serum potassium levels after finerenone initiation. Data are expressed as the mean (95% CI). ^*^P < .001, ^**^P < .0001 vs baseline.

Figure 6 shows the changes in serum potassium levels, which increased slightly after finerenone initiation but remained within the acceptable range during follow-up. The mean maximum serum potassium concentration during follow-up was 4.75 ± 0.48 mmol/L. Of note, three patients experienced hyperkalemia (serum potassium ≥5.5 mmol/L) requiring temporary discontinuation of finerenone. There were no cases of life-threatening hyperkalemia or hospitalizations due to acute kidney injury, hyperkalemia, ESKD or requirement for kidney replacement therapy during the follow-up period. Finerenone was well tolerated, and none of the patients developed serious AEs such as symptomatic hypotension, acute kidney injury, heart failure or cardiovascular events.

## DISCUSSION

In this real-world study, finerenone was shown to significantly reduce albuminuria and improve the eGFR slope in patients with DKD, who had residual albuminuria, regardless of baseline eGFR or albuminuria levels, despite treatment with RAS inhibitors. Finerenone improved the rate of GFR decline regardless of whether it was administered concomitantly with an SGLT2 inhibitor. Finerenone was well tolerated and treatment could be continued without notable hyperkalemia. Systolic and diastolic blood pressures were slightly but significantly reduced. Furthermore, finerenone reduced urinary β2MG and NAG levels as well as the NT-proBNP concentration in patients with DKD.

Improvement in kidney hard outcomes, such as progression to ESKD, may represent the most meaningful clinical benefit, but assessment of the eGFR slope, a surrogate outcome for ESKD, provides valuable insights. Changes in eGFR slope are strongly associated with kidney hard outcomes, allowing for analysis of data from all study participants [14]. Because the study duration was 12 months, it was not feasible to observe kidney hard outcomes, especially in patients with preserved kidney function or microalbuminuria. Therefore, to better evaluate the impact of finerenone on kidney function, we used eGFR slope as the primary endpoint.

In the FIDELIO-DKD trial [10], which primarily targeted patients with DKD who had albuminuria, finerenone reduced the risk of the primary renal composite endpoint (ESKD, a sustained decline in eGFR of ≥40%, and renal death) by 18% compared with placebo (*P* = .0014). In terms of exploratory endpoints, finerenone significantly reduced the UACR by 31% between baseline and 4 months after treatment compared with placebo, and this reduction was maintained beyond 4 months. In the FIGARO-DKD (Finerenone in Reducing Cardiovascular Mortality and Morbidity in Diabetic Kidney Disease) trial [11], which targeted patients with relatively early DKD, approximately half of the subjects had CKD stages G1–G2 or microalbuminuria. Although there was no significant difference in the secondary renal composite endpoint, the rate tended to be lower with finerenone than with placebo (hazard ratio 0.87; 95% CI 0.76, 1.01; *P* = .0689). In the FIGARO-DKD trial, the change in UACR between baseline and 4 months after treatment was significantly reduced by 32% in the finerenone group compared with the placebo group. Therefore, finerenone is expected to reduce the UACR regardless of CKD stage. In the present study, which included patients with eGFR categories G1–G4, the UACR decreased significantly by 37.7%, 44.2% and 49.1% at 3, 6 and 12 months, respectively, after initiating finerenone. The 2024 American Diabetes Association recommends a reduction in the UACR of ≥30% in patients with DKD and a UACR of ≥300 mg/gCr to slow CKD progression [15]. *Post hoc* analyses of the FIDELIO-DKD and FIGARO-DKD trials revealed that a reduction in UACR of >30% mediated 84% and 37% of the treatment effects on kidney and cardiovascular outcomes, respectively [16]. Therefore, the reduction in UACR observed after finerenone treatment in the present study may favorably impact future progression of cardiovascular and kidney diseases.

In one study, finerenone was administered for 90 days and demonstrated a dose-dependent decrease in UACR [17]. The final dose of finerenone in the FIDELIO-DKD trial was 15.1 mg, whereas in the present study it was 19.0 mg, with 90% of patients reaching the maximum dose of 20 mg [10]. Furthermore, when comparing the 10 mg and 20 mg groups, the 20 mg group had a significantly lower UACR. Patients who maintained a daily finerenone dose of 10 mg showed no significant decline in eGFR, but their serum potassium levels remained at 4.9–5.4 mmol/L, making it impossible to increase the finerenone dose up to 20 mg. Therefore, maximizing the dose of finerenone may be useful to reduce cardiac and renal endpoints unless there is hyperkalemia or a severe reduction of eGFR. However, further research is needed to determine whether these patients should receive increased finerenone doses with increased dietary potassium restriction or with concomitant use of potassium binders.

The results of this study demonstrate that finerenone improved the decline of the eGFR slope regardless of whether it was administered concomitantly with an SGLT2 inhibitor, but no significant difference was observed in terms of reduction in the UACR ratio. The FIDELIO-DKD [10] and FIGARO-DKD [11] trials were initiated before the efficacy of SGLT2 inhibitor therapy for DKD was established, and the rate of concomitant use of SGLT2 inhibitor therapy was low at 6.7%. In contrast, the present study examined the efficacy and safety of adding finerenone in accordance with the latest guidelines, and the rate of use of SGLT2 inhibitor therapy at baseline was 76.7%. The findings of this study reflect actual clinical practice and demonstrate that finerenone can be used safely regardless of whether an SGLT2 inhibitor is used concomitantly. In the CONFIDENCE (Combination Effect of Finerenone and Empagliflozin in Participants with Chronic Kidney Disease and Type 2 Diabetes Using a Urinary Albumin-to-Creatinine Ratio Endpoint) trial, coadministration of finerenone and empagliflozin in patients with DKD resulted in an early and additive reduction in UACR, with a 52% reduction from baseline at 180 days [18]. The decline in UACR with finerenone monotherapy and empagliflozin monotherapy was 29% and 32%, respectively, significantly lower than with the combination. In the CONFIDENCE trial, the nadir of the initial dip in eGFR was observed at Day 14 with empagliflozin monotherapy, whereas the nadir was observed at Month 3 with finerenone monotherapy. This trajectory was similar to that in our study, with the eGFR also reaching its nadir 3 months after starting on finerenone. Although the CONFIDENCE trial was short (i.e. 6 months), the nadir in UACR was observed at 180 days in both groups, whereas in our study, the reduction in UACR was sustained through to Month 12.

In our study, finerenone administration achieved small but significant reductions in systolic and diastolic blood pressures. A report using the FIDELIO-DKD trial dataset [10] found that blood pressure was reduced significantly by 3.84 mmHg in the finerenone group in comparison with the placebo group after 4 months in the overall population. However, when grouped according to quartile, the systolic blood pressure at baseline was positively correlated with the antihypertensive effect. Other studies in which baseline systolic blood pressure was well controlled at 126.9 mmHg or 127.7 mmHg did not show a significant reduction in blood pressure [19, 20]. In contrast, the baseline systolic blood pressure in the present study was 133 mmHg, suggesting that finerenone has a mild blood pressure–lowering effect.

The MR is expressed in cardiovascular tissues and in the kidney, and its excessive activation leads to cardiovascular disorders such as heart failure, myocardial fibrosis and coronary artery disease, and to albuminuria and fibrosis in the kidney, resulting in renal damage [21]. While the classical mechanism of MR activation is in an aldosterone-dependent pathway mediated by the renin–angiotensin–aldosterone system, the existence of RAS-independent pathways triggered by hypertension, obesity, and excessive salt intake has recently emerged [21, 22]. Furthermore, hyperglycemia has been reported to decrease MR degradation and increase sensitivity to MR [23, 24]. Moreover, the pathology of DKD, in which sodium accumulation and hyperglycemia overlap, is of concern because it may enhance activation of the MR. Therefore, while RAS inhibitors alone cannot suppress MR overactivation, MRAs, which act directly on the MR to suppress its overactivation, are promising therapeutic options for DKD. It has been suggested that the MRA finerenone inhibits the onset and progression of cardiovascular and renal disorders by suppressing inflammation and fibrosis caused by excessive activation of MRs. Significant decreases in urinary β2MG and NAG were observed in the present study. Therefore, finerenone may alleviate not only glomerular damage but also tubulointerstitial damage. Steroidal MRAs, such as spironolactone and eplerenone, have both cardiac and kidney benefits, but their widespread use has been associated with increased hyperkalemia [25]. In contrast, the nonsteroidal MRA finerenone offers similar benefits while minimizing the risk of hyperkalemia [26]. In the FIDELITY (Finerenone in chronic kidney disease and type 2 diabetes: Combined FIDELIO-DKD and FIGARO-DKD trial programme analysis) analysis, AEs related to hyperkalemia occurred in 6.9% of the placebo group and 14.0% of the finerenone group. The incidence of these events leading to discontinuation of the study drug was higher in the finerenone group (0.6% vs 1.7%) [27]. In addition, the frequency of hyperkalemia was lower when finerenone was administered in combination with an SGLT2 inhibitor was than when it was administered alone [28]. In the present study, serum potassium levels increased slightly but remained within acceptable limits, and the incidence of hyperkalemia was low. The low incidence of hyperkalemia-related AEs is consistent with real-world studies reporting similar hyperkalemia rates (2 to 3 cases per 100 patients) [7], and no hospitalizations attributable to hyperkalemia were reported. However, serum potassium, eGFR and UACR values should be closely monitored during regular follow-up.

The strengths of this study include its real-world setting, which includes a wider range of kidney function and albuminuria levels than would be possible in a clinical trial, making the results more relevant to daily clinical practice. Furthermore, approximately 77% of this cohort were on SGLT2 inhibitor therapy, which is uncommon in clinical trials. Our analysis of improvement in the eGFR slope provides new insights into early predictors of treatment response.

This study also has several limitations. First, it was performed at a single center with a small sample size (*n* = 120). Second, it had a retrospective observational design with a short follow-up period of 12 months. This study examined only short-term measurements of eGFR slope as renal outcomes, and did not suggest any effect on improving long-term renal outcomes, such as initiating kidney replacement therapy, achieving eGFR of <15 mL/min/1.73 m^2^, or achieving a sustained eGFR decline of ≥40%. Third, approximately 77% of the study participants were taking SGLT2 inhibitors, which have not been commonly used in large-scale clinical trials. These differences may limit the comparability of the results of this study with those of large-scale clinical trials and highlight the need for further research to clarify the optimal combination and sequence of treatments for patients with DKD. Finally, no comparison was made between patients who received finerenone and those who did not. Matching of background factors in a retrospective observational study comparing finerenone-treated and finerenone-non-treated groups of patients with DKD would be difficult and potentially lead to confounding bias. Therefore, a further prospective, double-arm, long-term, multicenter study with a larger sample size is needed to clarify the cardiovascular and kidney composite endpoint of finerenone in clinical practice.

In conclusion, this analysis of real-world data suggests that finerenone may reduce albuminuria and improve the eGFR slope in patients with DKD in the absence of significant hyperkalemia, regardless of baseline eGFR and albuminuria values. Finerenone reduced urinary β2MG and NAG levels, suggesting that it may contribute to preventing tubulointerstitial damage. Furthermore, finerenone significantly reduced blood pressure and NT-proBNP levels, albeit slightly, suggesting that it may have protective effects not only on the kidney but also on the heart in DKD patients. These findings suggest that finerenone may be a useful treatment option in the real-world setting. However, to clarify the hard kidney outcomes of finerenone in clinical practice, a prospective, double-arm, long-term, multicenter study with a larger sample size is needed.

## Data Availability

The data underlying this article will be shared by the corresponding author upon reasonable request.
